# Identification of osteoporosis based on gene biomarkers using support vector machine

**DOI:** 10.1515/med-2022-0507

**Published:** 2022-07-07

**Authors:** Nanning Lv, Zhangzhe Zhou, Shuangjun He, Xiaofeng Shao, Xinfeng Zhou, Xiaoxiao Feng, Zhonglai Qian, Yijian Zhang, Mingming Liu

**Affiliations:** Department of Orthopedic Surgery, The Second People’s Hospital of Lianyungang, Lianyungang, Jiangsu 222003, China; Department of Orthopedic Surgery, The First Affiliated Hospital of Soochow University, Suzhou, Jiangsu 215000, China; Department of Orthopedic Surgery, Affiliated Danyang Hospital of Nantong University, The People’s Hospital of Danyang, Danyang, Jiangsu 212300, China

**Keywords:** osteoporosis, differentially expressed genes, weighted gene co-expression network analysis, protein–protein interaction, support vector machine

## Abstract

Osteoporosis is a major health concern worldwide. The present study aimed to identify effective biomarkers for osteoporosis detection. In osteoporosis, 559 differentially expressed genes (DEGs) were enriched in PI3K-Akt signaling pathway and Foxo signaling pathway. Weighted gene co-expression network analysis showed that green, pink, and tan modules were clinically significant modules, and that six genes (VEGFA, DDX5, SOD2, HNRNPD, EIF5B, and HSP90B1) were identified as “real” hub genes in the protein–protein interaction network, co-expression network, and 559 DEGs. The sensitivity and specificity of the support vector machine (SVM) for identifying patients with osteoporosis was 100%, with an area under curve of 1 in both training and validation datasets. Our results indicated that the current system using the SVM method could identify patients with osteoporosis.

## 1 Introduction

Osteoporosis is a systemic metabolic disease characterized by a decrease in bone mass and destruction of bone tissue microstructure, which could lead to bone fragility and susceptibility to fracture. As a common bone disease syndrome [1], osteoporosis has been considered as an aging disease in which bone density decreases and further aggravates the condition [2,3]. In normal physiological state, human bones are in constant metabolism that osteoblasts form new bone tissue and osteoclasts resorb old or damaged bone tissue. The dynamic balance of bone volume is maintained by continuous bone formation and bone resorption [4]. However, when the body is affected by injury, aging, estrogen deficiency, and/or other factors, the balance will be disrupted, causing osteoporosis [5,6].

As a supervised learning method in machine learning, support vector machine (SVM) algorithm [7] was first proposed in 1995, and has been developed more maturely and widely applied in the mid-1990s. Later on, a series of improved and extended algorithms, including multi-classification SVM, support vector regression, least squares SVM, support vector clustering, semi-supervised SVM, etc., have been developed.

Machine learning tools could display key features from complex datasets [8,9]. SVM is widely used in disease research for building predictive models, and could produce effective and predictable models [10–12]. For example, a study has shown that applying SVM approach helps identify postmenopausal women with low bone density [13]. Moreover, a gene signature associated with postmenopausal osteoporosis was developed and validated by SVM [14]. Chen et al. reported that a diagnostic model established based on nine key genes could reliably separate osteoporosis patients from healthy subjects [15]. Chen et al. have identified a group of circulating miRNAs as non-invasive biomarkers for the detection of postmenopausal and mechanical unloading osteoporosis through a large-scale screening based on microarray [16]. Xia et al. showed that genes such as VPS35, FCGR2A, TBCA, HIRA, TYROBP, JUND, PHF20, NFKB2, RPL35A, and BICD2 may be considered to be potential pathogenic genes of osteoporosis and may be useful for further study of the mechanisms underlying osteoporosis [17]. Although a large number of biomarkers have been identified in different ways, there is still a lack of model analyses based on multiple key genes that are valid in osteoporosis for clinical application.

The Gene Expression Omnibus (GEO) [18], an online public database made available by National Center for Biotechnology Information (NCBI) in 2000, is currently one of the most comprehensive gene expression databases. We systematically analyzed the expression patterns of genes associated with osteoporosis samples from this database at a transcriptional level. In addition, a risk prediction model was developed for osteoporosis patients based on SVM.

## 2 Materials and methods

### 2.1 Data acquisition

MINiML formatted family file(s) of osteoporosis-related microarray datasets GSE35959 [19], GSE7158 [20], GSE7429 [21], and GSE13850 (https://www.ncbi.nlm.nih.gov/geo/query/acc.cgi?acc=GSE13850) and GSE62402 (https://www.ncbi.nlm.nih.gov/geo/query/acc.cgi?acc=GSE62402) were downloaded from GEO.

From the GEO dataset, osteoporosis samples and control samples were retained. The probes were converted to Gene Symbol. One probe corresponding to multiple genes was removed. Median expressions of genes corresponding to multiple Gene Symbols were taken. The clinical statistics of the processed samples are shown in Table 1.

**Table 1:** Clinical information of the samples

Dataset	Expression	Platforms
**GSE35959**		
Control	14	GPL570
Osteoporosis	5	
**GSE7158**		
High PBM	14	GPL570
Low PBM	12	
**GSE62402**		
High BMD	5	GPL5175
Low BMD	5	
**GSE13850**		
High BMD	20	GPL96
Low BMD	20	
**GSE7429**		
High BMD	10	GPL96
Low BMD	10	

PBM (Peak bone mass) is an important determinant of osteoporosis. BMD (bone mineral density) can be reliably and accurately measured and has high genetic determination with heritability of 0.5–0.9, indicating that genetic factors play an important role in risk of osteoporosis.

### 2.2 Identification of differentially expressed genes (DEGs)

The “Limma” package [22] in the latest R language was used to screen DEGs between osteoporosis tissues and normal samples under the interception criteria |log2FC| > 1 and FDR < 0.05.

### 2.3 Functional enrichment analysis

Kyoto Encyclopedia of Genes and Genomes (KEGG) pathway analysis and gene ontology (GO) functional enrichment analysis (cellular component [CC], biological process [BP], and molecular function [MF]) on the DEGs were performed using the R software package clusterProfiler [23]. *p* < 0.05 was the threshold for significant enrichment.

### 2.4 Weighted gene co-expression network analysis (WGCNA)

WGCNA was performed on the DEGs using the R language “WGCNA” package [24]. The expression profile in the osteoporosis samples was constructed as a sample clustering dendrogram using the function hclust, and sample clustering analysis was performed. If the gene expression value of a sample was significantly higher than the average, the sample was considered as an outlier. The vertical coordinate was the height of the sample clustering tree, with a higher height indicating a higher gene expression of the sample. The soft threshold was selected using the function pick soft threshold, a criterion based on the approximate scale-free network that allows the constructed network to be more consistent with the power-law distribution. In this study, the correlation coefficient was 0.85.

Based on the topological overlap measure, the genes were clustered using the average linkage hierarchical clustering method under the criteria of hybrid dynamic shear tree. The minimum number of genes per gene network module was 100, and 0.25 was the threshold of cut height.

### 2.5 Protein–protein interaction (PPI) networks

PPI network could help discover new drug targets and study the molecular mechanisms of a disease from a system perspective. The PPI network was constructed based on the STRING database, which is a database used for searching interactions between known proteins and predicting protein interactions. The PPI network of the module genes was developed by importing the module genes into the STRING database. Generally, the importance of a network node is positively related to greater connections to the genes in the network. Four algorithms of Degree, Maximal Clique Centrality (MCC), Closeness, and Betweenness of Cytoscape (version: 3.7.2) [25] software plug-in cytoHubba [26] were used to calculate each point in the PPI network for screening the module pivot genes.

### 2.6 Construction of SVM classifier

SVM is a supervised machine learning classification algorithm that distinguishes sample types by estimating the degree of a sample belonging to a certain class. For the training set, the SVM classifier was constructed using the SVM method based on the optimal mRNA set in R package e1071 (version 1.6-8, http://cran.r-project.org/web/packages/e1071).

The performance of the SVM classifier was evaluated in the training and validation sets with the area under curve (AUC) of the receiver operating characteristic (ROC) curve as an evaluation metric.

GitHub page

https://github.com/wumark456/work

## 3 Results

### 3.1 Identification and functional annotation of DEGs

**Figure 1:**
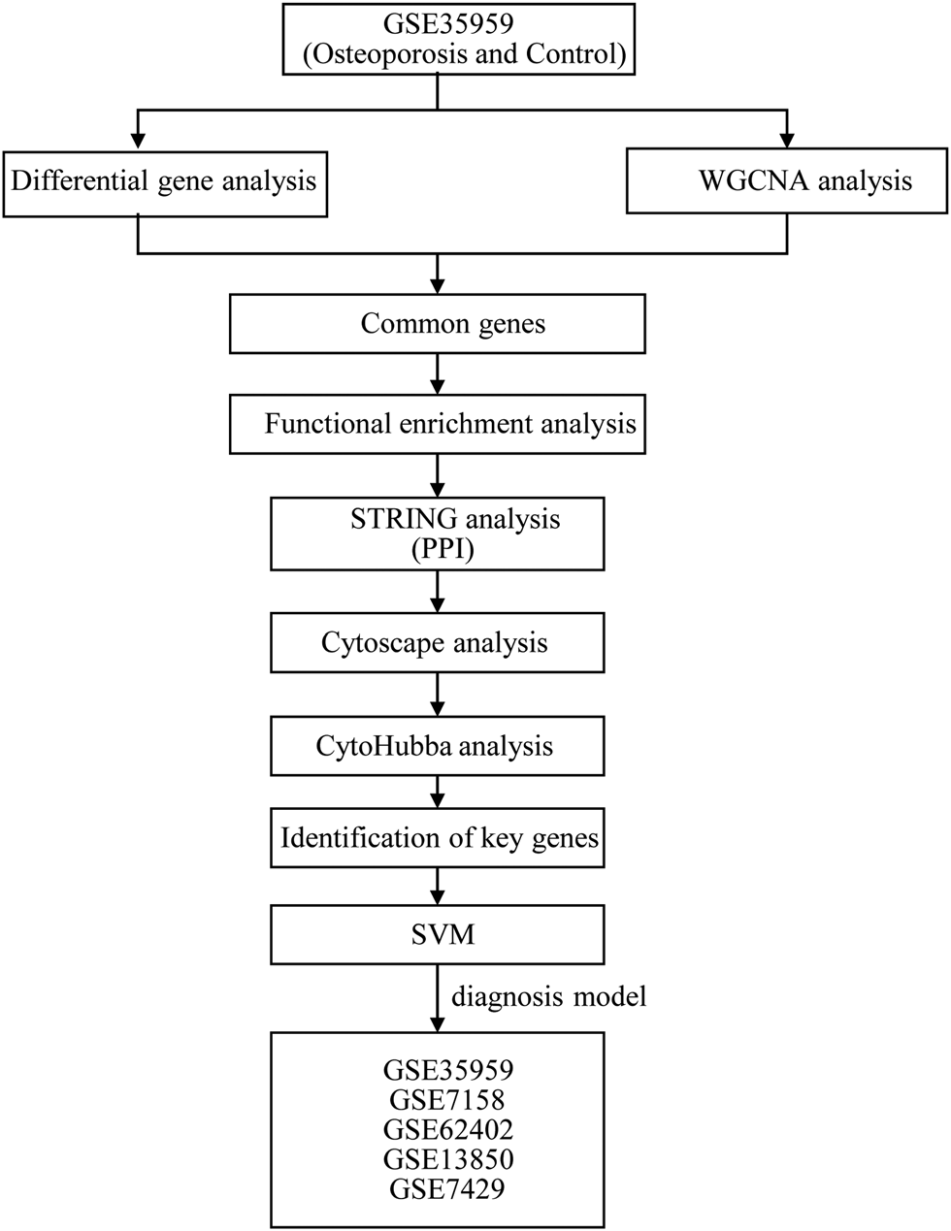
Work flow chart.

**Figure 2:**
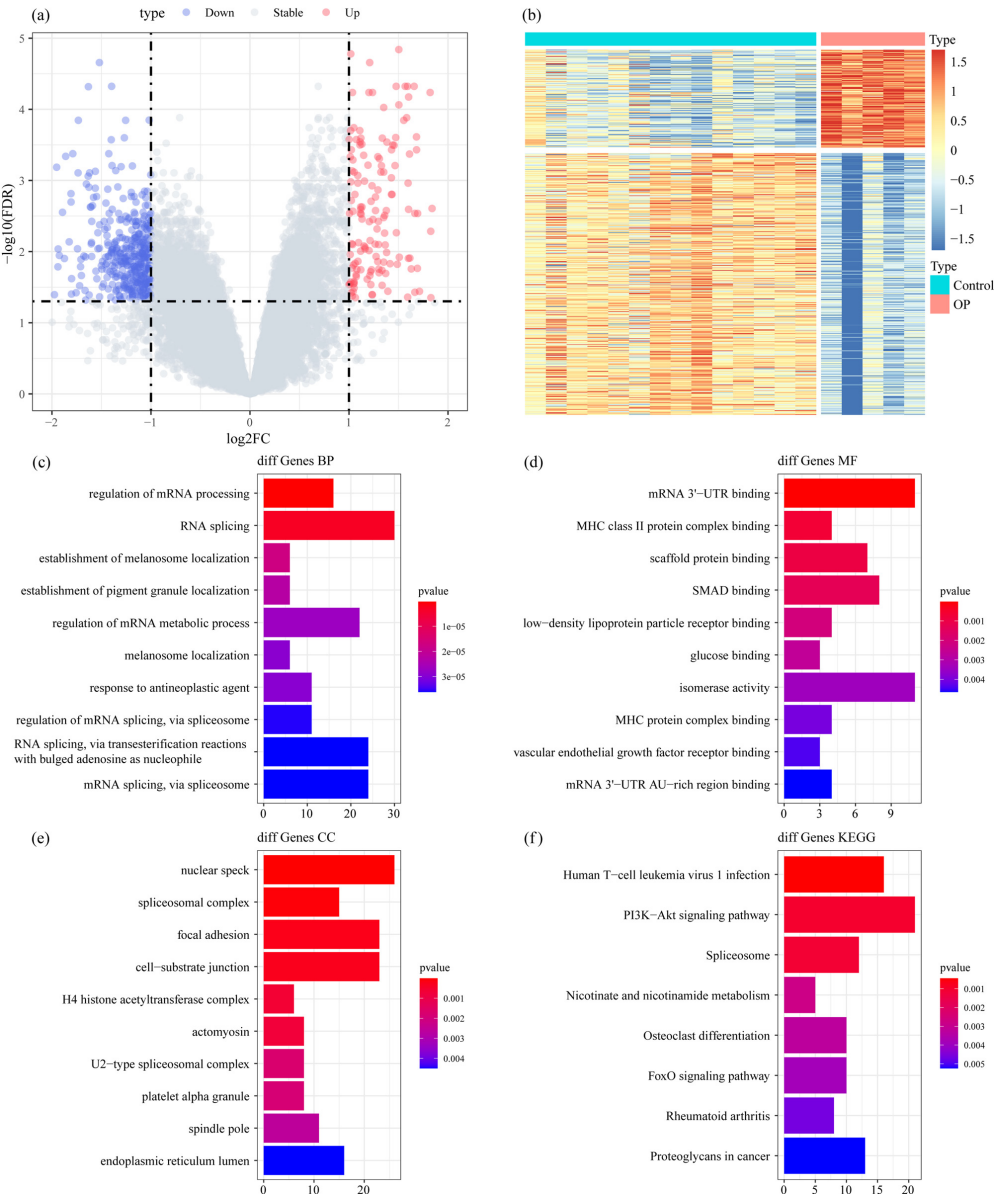
Identification of DEGs: (a) the volcano map of DEGs in GSE35959 dataset, (b) the heat map of DEGs in GSE35959 dataset, (c) the BP annotation map of DEGs, (d) the CC annotation map of DEGs, (e) the MF annotation map of DEGs, and (f) the KEGG annotation map of DEGs.

The analysis flow chart of this study is shown in Figure 1. The Limma package was used to calculate the DEGs between osteoporosis and control samples from GSE35959 dataset. A total of 559 DEGs were obtained incorporating 152 upregulated and 407 downregulated genes in osteoporosis samples (Figure 2a and b). Furthermore, KEGG pathway analysis and GO function enrichment analysis on the 559 DEGs were performed using R software package Clusterprofiler. The top ten significantly enriched BPs, MFs, and CCs are shown in Figure 2c–e. Based on the KEGG annotation, eight pathways including PI3K-Akt signaling pathway, Foxo signaling pathway, and osteoclast differentiation (Figure 2f) were obtained.

### 3.2 WGCNA

**Figure 3:**
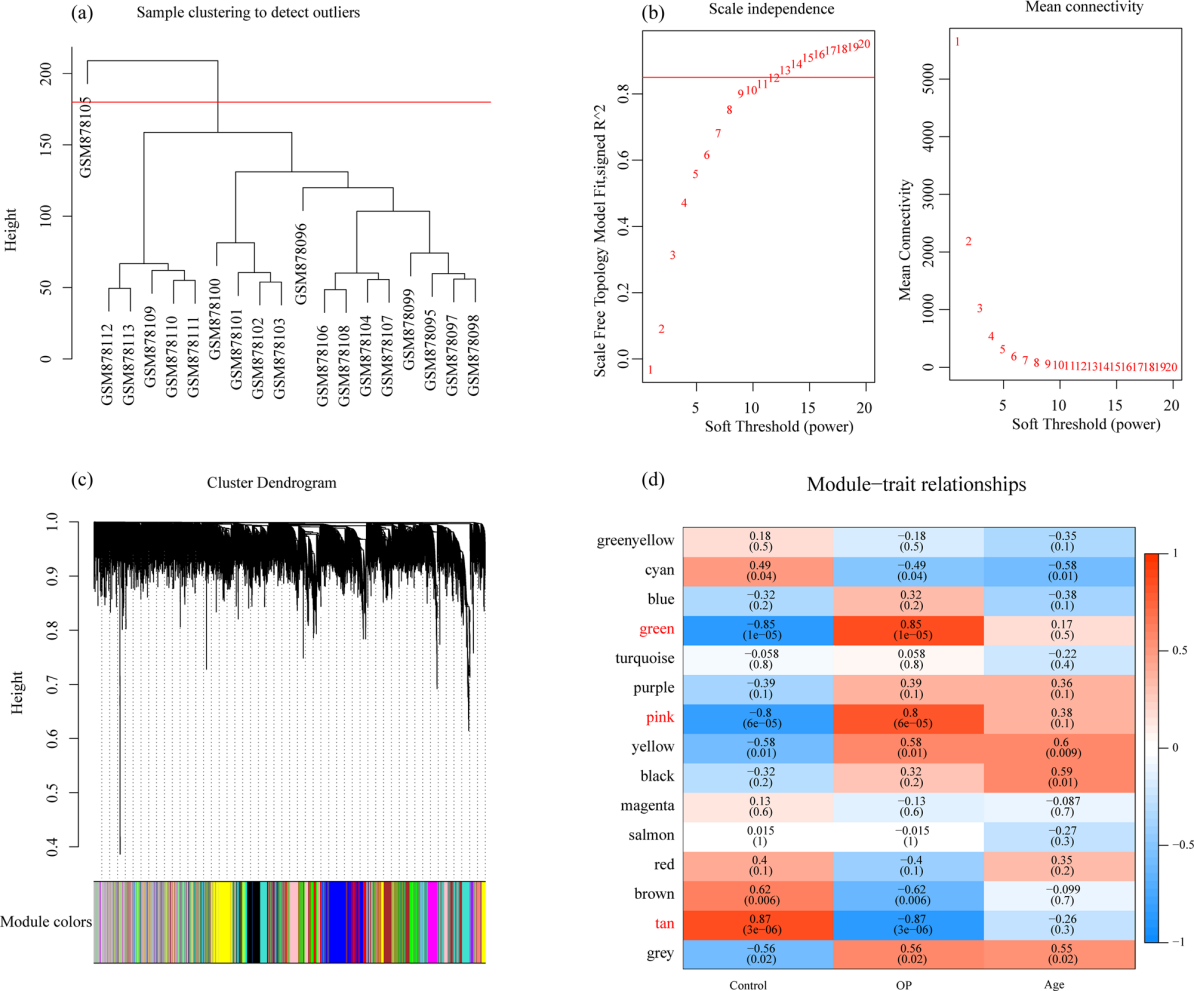
WGCNA: (a) cluster analysis of samples, (b) analysis of network topology for various soft-thresholding powers, (c) gene dendrogram and module colors, and (d) correlation results between 15 modules and clinical phenotypes.

**Figure 4:**
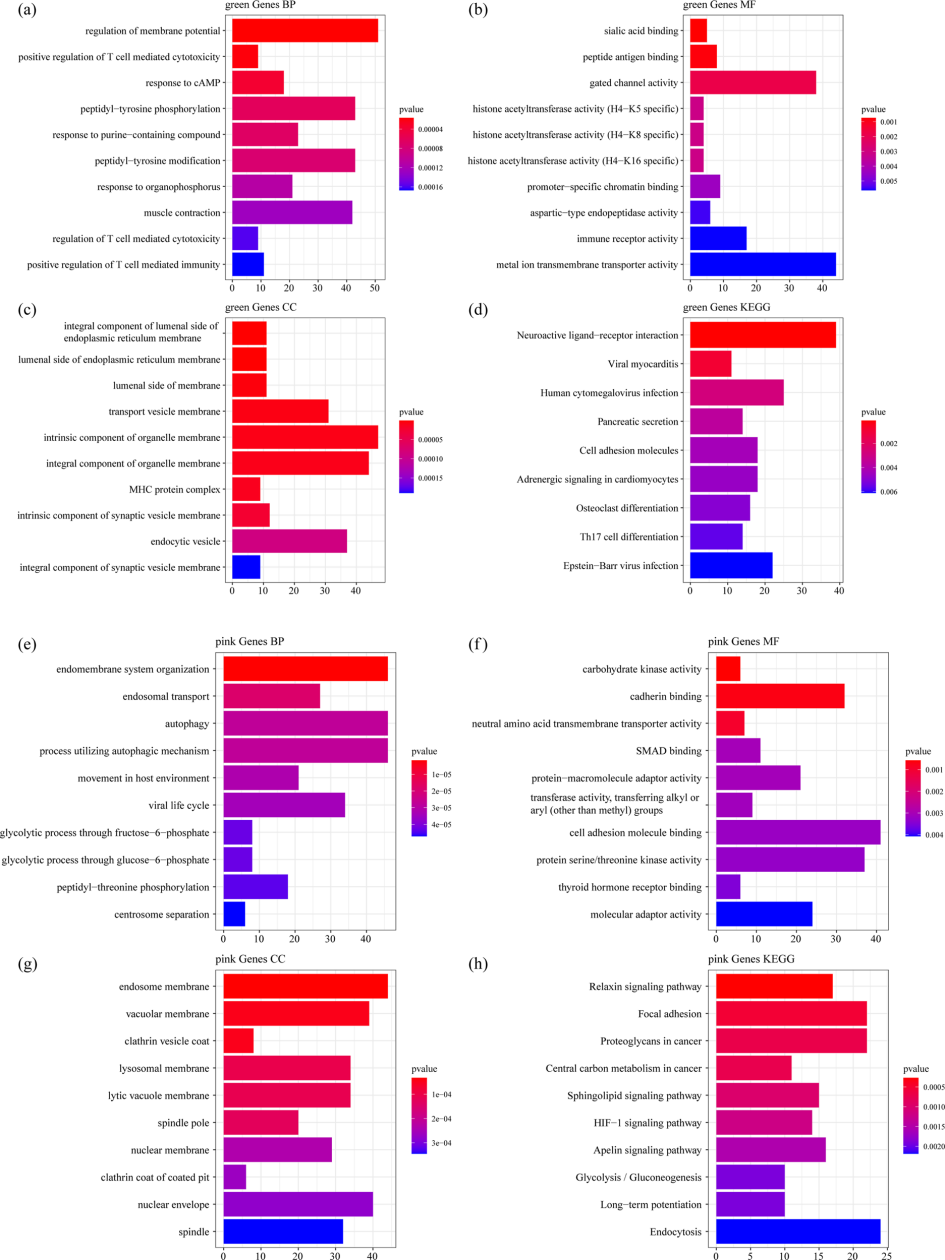
Functional enrichment analysis of DEGs in green and pink model: (a) the BP annotation map of DEGs in green model, (b) the CC annotation map of DEGs in green model, (c) the MF annotation map of DEGs in green model, (d) the KEGG annotation map of DEGs in green model, (e) the BP annotation map of DEGs in pink model, (f) the CC annotation map of DEGs in pink model, (g) the MF annotation map of DEGs in pink model, and (h) the KEGG annotation map of DEGs in pink model.

WGCNA was used to construct a co-expression network and identify co-expression modules. The hierarchical clustering of samples showed that 18 samples met the requirements (Figure 3a). The study of sufficient soft threshold validation converged to a scale-free topology with a value of 13 (Figure 3b). The first group of modules was obtained by Dynamic Tree Cut algorithm, and the related modules (height = 0.25, deepSplit = 2, and minModuleSize = 100) were merged together (merged Dynamic). Here, we identified a total of 15 modules (Figure 3c). The correlation between each module and the sample type (osteoporosis and control) was further analyzed. The results demonstrated that the green and pink modules were significantly positively correlated with osteoporosis, and that the tan module was obviously negatively correlated with osteoporosis (Figure 3d). Furthermore, using the R software package Clusterprofile, 1,719 genes, 1,143 genes, and 225 genes obtained, respectively, from green, pink, and tan modules of WGCNA co-expression were analyzed for KEGG pathway analysis and GO function enrichment. For the 1,719 genes in the green model, and the top ten significantly enriched BPs, MFs, and CCs are shown in Figure 4a–c. Based on KEGG annotation, nine pathways including osteoclast differentiation, cell adhesion molecules, Th17 cell differentiation, and Epstein-Barr virus infection were obtained (Figure 4d).

For the 1,143 genes in pink model, the top ten significantly enriched BPs, MFs, and CCs are shown in Figure 4e–g. Based on the KEGG annotation, 73 pathways including HIF-1 signaling pathway, Apelin signaling pathway, mTOR signaling pathway, and MAPK signaling pathway were obtained (Figure 4h).

**Figure 5:**
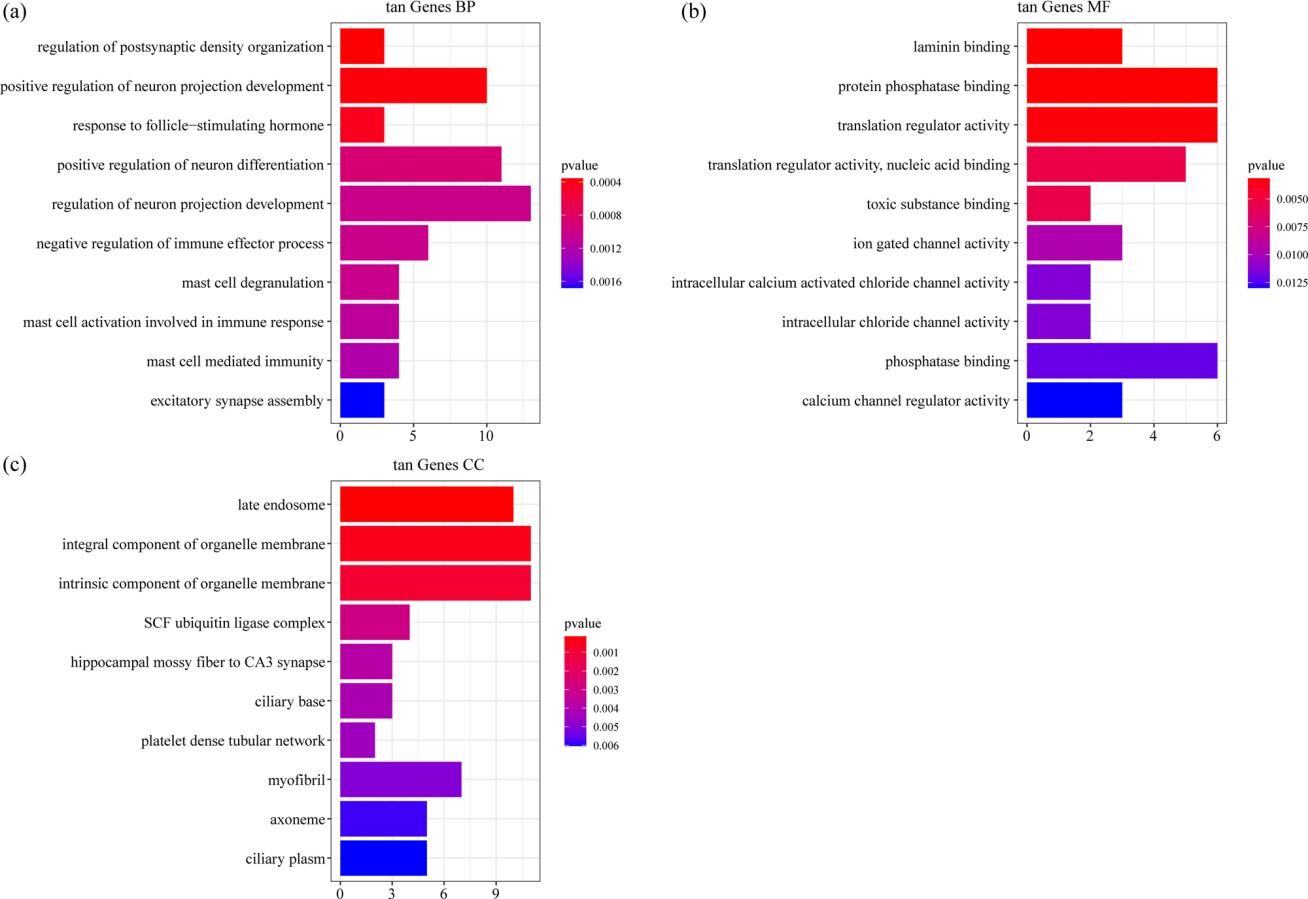
Functional enrichment analysis of DEGs in tan model: (a) the BP annotation map of DEGs in tan model, (b) the CC annotation map of DEGs in tan model, and (c) the MF annotation map of DEGs in tan model.

For the 225 genes in tan model, the top ten significantly enriched BPs, MFs, and CCs are shown in Figure 5a–c. There were no pathways obtained based on the KEGG annotation.

### 3.3 Identification of co-expressed differential genes

**Figure 6:**
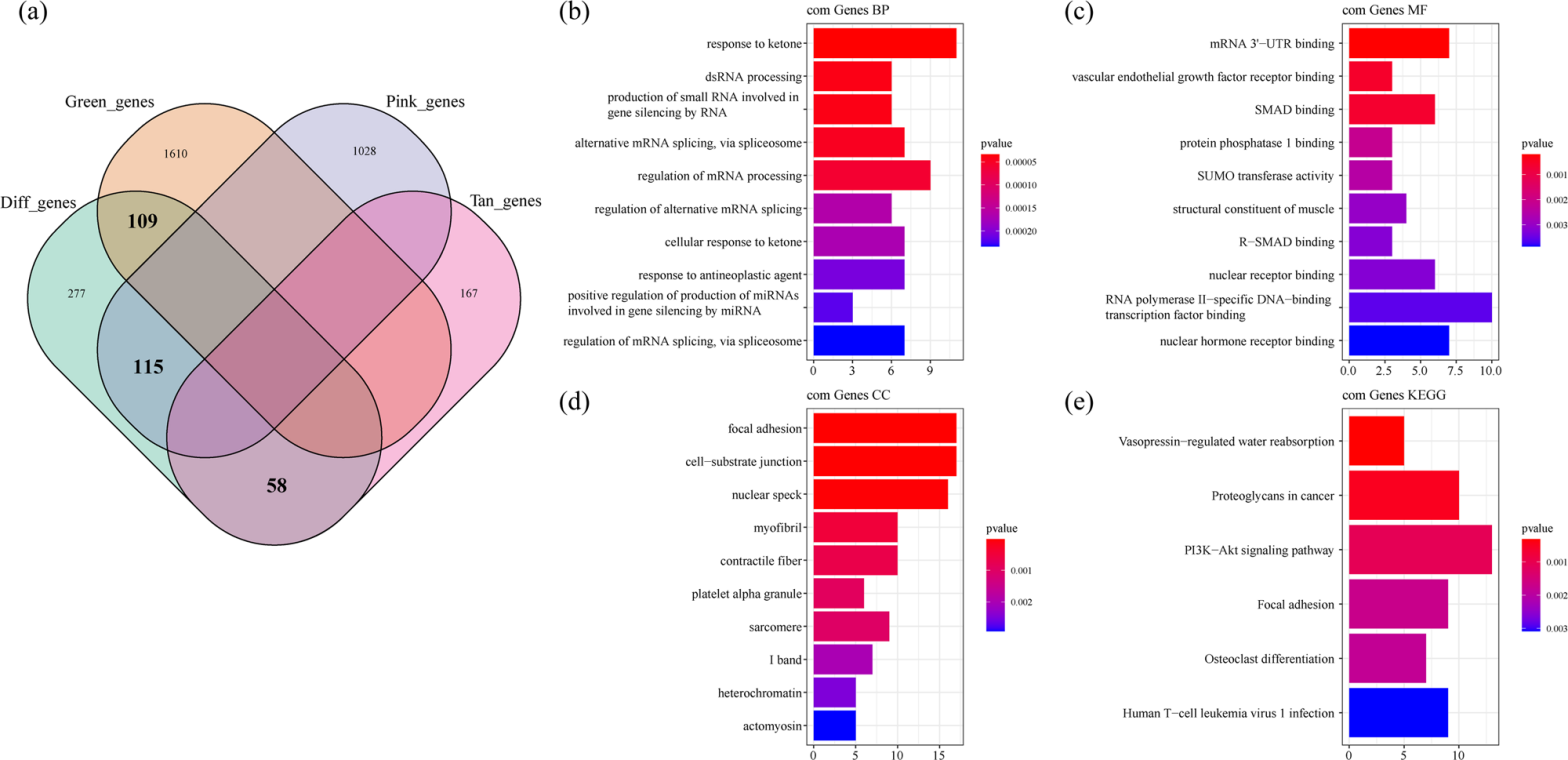
Identification of co-expression of DEGs: (a) Venn diagram of co-expressed genes and DEGs, (b) the BP annotation map of co-expression of DEGs, (c) the CC annotation map of co-expression of DEGs, (d) the MF annotation map of co-expression of DEGs, and (e) the KEGG annotation map of co-expression of DEGs.

The DEGs in GSE35959 dataset and green, pink, and tan co-expressed by WGCNA showed 109 intersected genes between the green module and 559 DEGs. Among the 559 DEGs, the pink module gene had 115 intersected genes, 58 intersected genes were obtained between the tan module and 559 DEGs, and a total of 282 genes were in the intersection (Figure 6a). The KEGG pathway analysis and GO function enrichment analysis on the 282 DEGs were performed by software package Clusterprofiler. The top ten significantly enriched BPs, MFs, and CCs are shown in Figure 6b–d. Based on the KEGG annotation, six pathways including PI3K-Akt signaling pathway, osteoclast differentiation, and focal adhesion were obtained (Figure 6e).

### 3.4 PPI network

**Figure 7:**
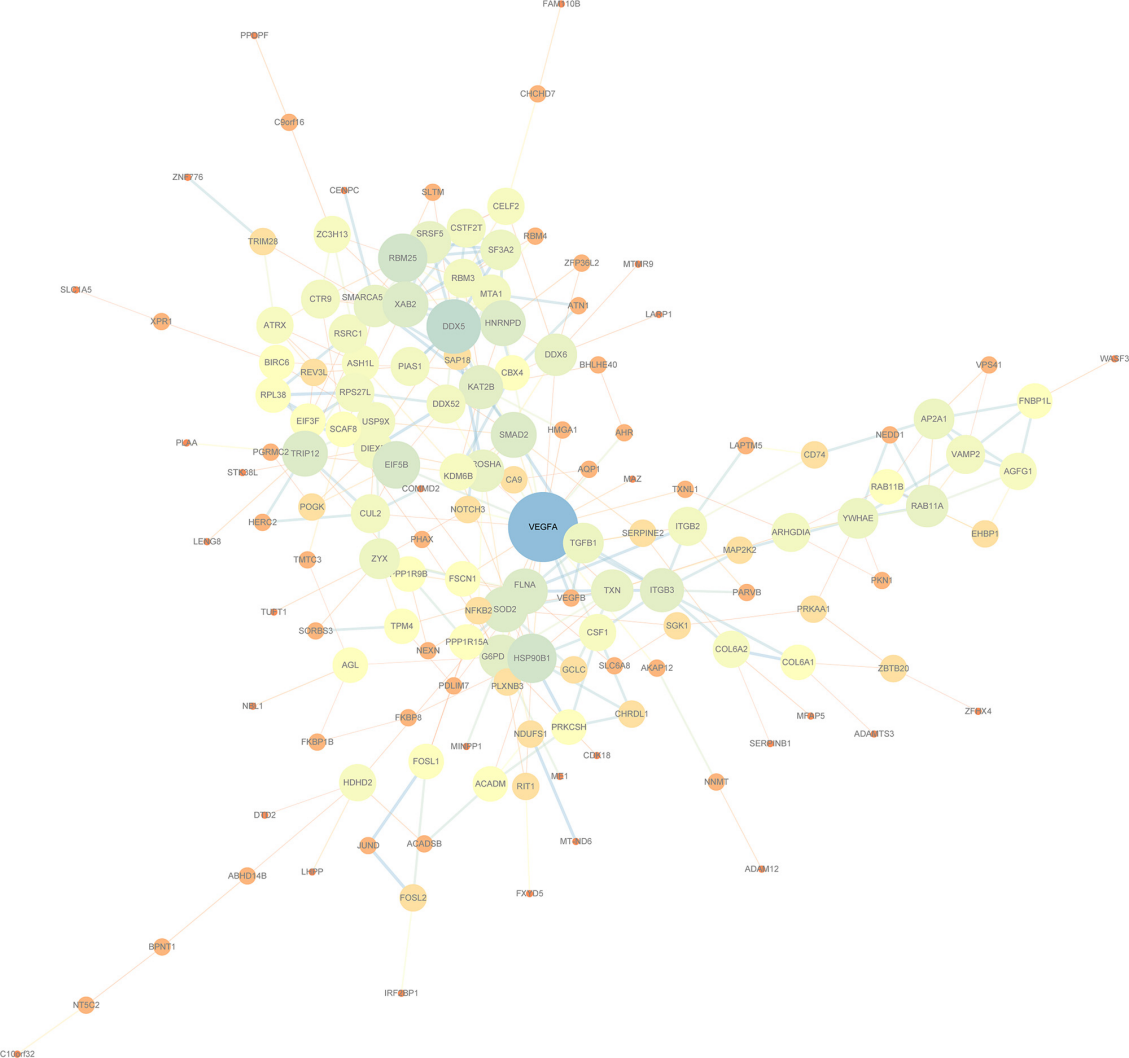
PPI diagram of STRING protein network visualized by Cytoscape.

**Figure 8:**
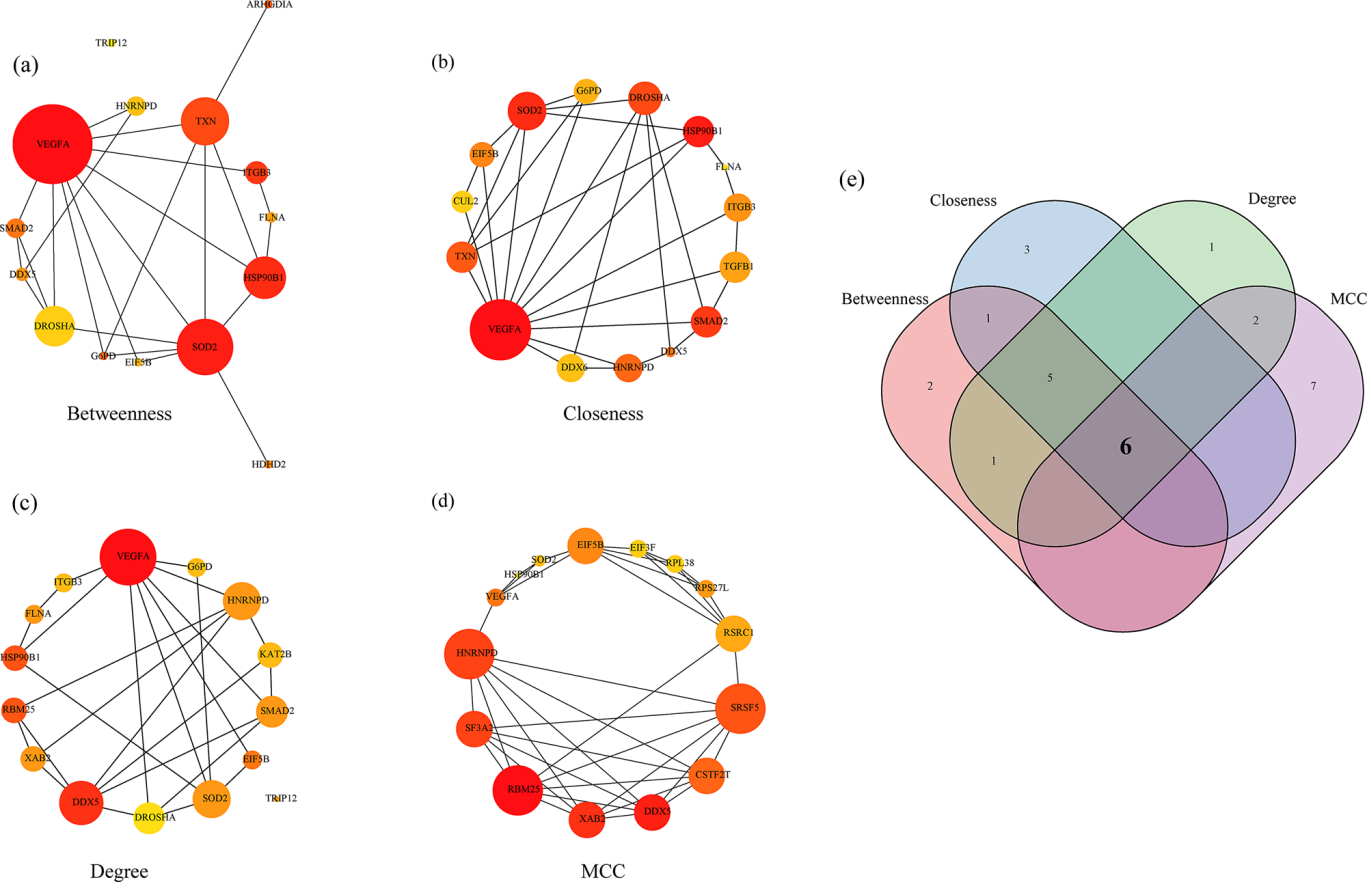
Identification of Hub genes: (a) PPI network of hub genes obtained by Betweenness algorithm, (b) PPI network of hub genes obtained by Closeness algorithm, (c) PPI network of hub genes obtained by Degree algorithm, (d) PPI network of hub genes obtained by MCC algorithm, and (e) Venn diagram of the Hub genes obtained by the four algorithms.

The PPI network of the 282 DEGs was further developed using the STRING database (Figure 7). For the PPI network developed with the 282 DEGs, the four algorithms of Degree, MCC, Closeness, and Betweenness of cytoHubba, a plug-in of Cytoscape (version: 3.7.2) software were used for calculations. Here, the top 15 genes were selected as the key genes (Figure 8a–d). The hub genes obtained by the four algorithms were taken as intersection, and finally six genes (*VEGFA, DDX5, SOD2, HNRNPD, EIF5B*, and *HSP90B1*) were acquired (Figure 8e).

### 3.5 Construction and validation of the diagnostic model

**Figure 9:**
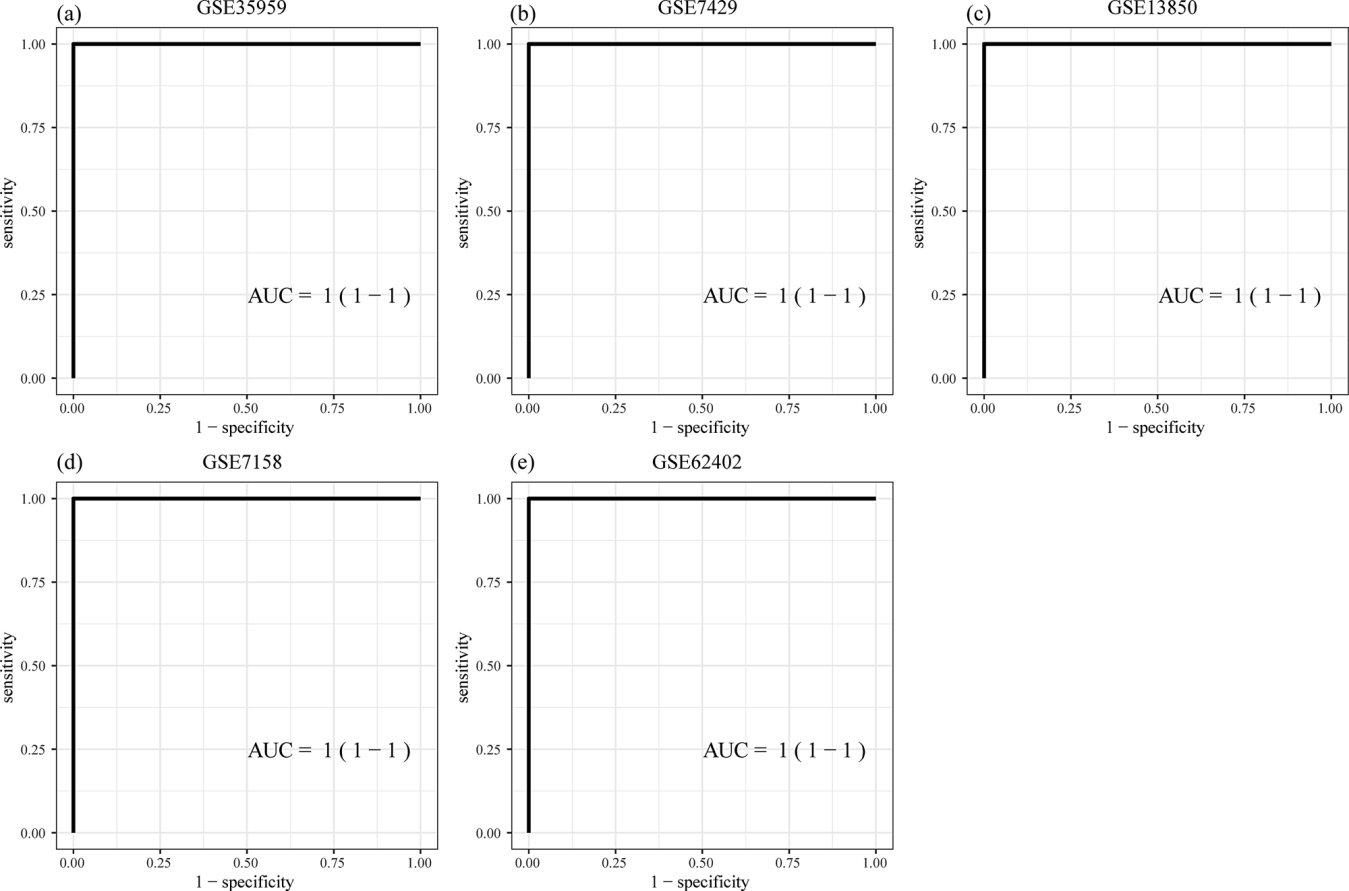
Construction and validation of diagnostic models: (a) ROC curves of the classification results of the diagnostic model on sample of GSE35959 dataset, (b) ROC curves of the classification results of the diagnostic model on the samples of GSE7429 dataset, (c) ROC curves of the classification results of the diagnostic model on the samples of GSE13850 dataset, (d) ROC curves of the classification results of the diagnostic model on the samples of GSE7158 dataset, and (e) ROC curves of the classification results of the diagnostic model on the samples of GSE62402 dataset.

GSE35959 was used as train dataset, and datasets (GSE7429, GSE7158, GSE13850, and GSE62402) were used as validation datasets. Six hub genes served as features in the training dataset, their corresponding expression profiles were obtained, and the SVM classification model was constructed. The classification accuracy of GSE35959 dataset was 100%, as 19 out of 19 samples were correctly classified; moreover, the sensitivity and specificity of the models were all 100%, with the area under the ROC curve (AUC) of 1 (Figure 9a). Furthermore, the GSE7429, GSE7158, GSE13850, and GSE62402 datasets verified that all the samples in the dataset were correctly classified. The classification accuracy was 100%, and the sensitivity and specificity of the models were 100%, with an area under the ROC curve of 1 (Figure 9b–e).

## 4 Discussion

Osteoporotic fractures can be prevented by pharmacological treatment. The current available osteoporosis treatments are anti-resorptive (inhibiting the osteoclasts), bone forming (stimulating the osteoblasts), or dual acting (simultaneously stimulating the osteoblasts and inhibiting the osteoclasts) [27]. The anti-resorptive treatments are bisphosphonates, receptor activator of nuclear factor κ-B ligand (RANKL) antibody, and selective estrogen receptor modulators (SERMs) that either cause osteoclast apoptosis (bisphosphonates) or inhibit osteoclast recruitment (RANKL antibody and SERMs). Teriparatide (parathyroid hormone, amino acids 1–34) and abaloparatide are bone-forming treatments of which abaloparatide currently is only available in the United States [27]. Romosozumab is a dual-acting treatment that stimulates bone formation and inhibits bone resorption at the same time [28].

In the present study, 559 DEGs between osteoporosis patients and normal controls were identified from GSE35959 dataset. WGCNA identified the green, pink, and tan modules as clinically significant. Subsequent analysis revealed six genes (*VEGFA*, *DDX5*, *SOD2*, *HNRNPD*, *EIF5B*, and *HSP90B1*) overlapped in PPI, 559 DEGs and co-expression analysis were the “real” hub genes. Based on SVM, the six-gene signature achieved a 100% prediction accuracy in distinguishing patients with osteoporosis from normal controls, with 100% sensitivity, 100% specificity, and AUC of 1. The results obtained using other four datasets (GSE7158, GSE62402, GSE13850, and GSE7429) further supported such findings.

SVM methods have the feasibility of extracting higher order statistics, and have been widely used in classification and prediction. Lin et al. used different classifiers to evaluate fractures from X-ray images, and reported a high classification accuracy of 98.2% based on a combination of SVM classifiers [29]. Caligiuri et al. showed that SVM is highly effective in distinguishing healthy bones with high Az values from fractured bones [30]. Based on the SVM, the 11-gene combination achieved a 94% prediction accuracy in distinguishing patients with postmenopausal osteoporosis from healthy controls [14]. Chen et al. identified six hub genes as features to build a predictive prognostic model for osteoporosis [31]. Hu et al. constructed a five-feature gene model by SVM for classifying osteoporosis samples [32]. Liu et al. screened three key pathways associated with the development of osteoporosis [33]. In this work, SVM was also applied to identify osteoporosis patients from normal samples based on a six-gene signature with 100% sensitivity.

As for VEGFA, Yu et al. reported that miR-16a-5p and VEGFA contributed to the postmenopausal osteoporosis [34]. A study demonstrated that in the osteoporosis group, VEGFA gene showed a significant association with osteoporosis [35]. VEGFA is a pro-angiogenic factor upregulated when responding to uniaxial cyclic tensile strain in human adipose-derived stem cells (hASCs) and hMSCs from osteoporotic donors [36]. DDX5 is differentially expressed in bone marrow microenvironment of osteoporosis patients [37]. Melatonin promotes osteogenesis by inducing oxidative stress in mitochondria via the SIRT3/SOD2 signaling pathway [38]. Deng et al. reported that SOD2 is a susceptibility gene for osteoporosis among Chinese population [39]. HnRNPL inhibits osteogenic differentiation of PDLCs through downregulating the H3K36me3-specific methyltransferase Setd2. However, there are no reports of HNRNPD in osteoporosis. According to previous study, EIF5B and HSP90B1 have also not been reported in osteoporosis. Still, further research is required to explore the roles of six genes in osteoporosis.

In summary, the present study applied weighted gene co-expression analysis combined with PPI analysis to identify hub genes associated with osteoporosis. In addition, the six-gene combinations may serve as potential biomarkers for osteoporosis to guide clinical treatment for different patients. However, lack of biological investigation and experimental validation with a larger sample size was considered as a limitation of this study. Further studies are therefore needed to verify the diagnostic ability of this gene signature for osteoporosis before clinical application.
